# Analysis of Anesthesia Methods in Percutaneous Kyphoplasty for Treatment of Vertebral Compression Fractures

**DOI:** 10.1155/2020/3965961

**Published:** 2020-01-07

**Authors:** Jie Liu, Lin Wang, Mei Chai, Junjie Kang, Jie Wang, Yanjun Zhang

**Affiliations:** ^1^Department of Anesthesia, Second Affiliated Hospital of Dalian Medical University, Dalian, China; ^2^School of Software Technology, Dalian University of Technology, Dalian, China

## Abstract

**Aim:**

Percutaneous kyphoplasty (PKP) is a routine operation for the treatment of vertebral compression fracture (VCF). Both local anesthesia and general anesthesia are widely used for PKP. However, which type of anesthesia is better for PKP still remains uncertain. This study aimed to find out whether local anesthesia or general anesthesia is more suitable for PKP.

**Methods:**

This is a retrospective clinical trial. A total of 85 single-level VCF patients who received PKP 12 months ago were recruited in this study. 45 patients who received local anesthesia were in group L, and 40 patients with general anesthesia were in group G. Clinical, radiological, and economic data between the two groups were collected.

**Results:**

No difference was found on preoperative data between the two groups. The duration of operation time in group L was longer than that in group G. Within 12 months after PKP, more complications happened in group G than those in group L.

**Results:**

No difference was found on preoperative data between the two groups. The duration of operation time in group L was longer than that in group G. Within 12 months after PKP, more complications happened in group G than those in group L.

**Conclusion:**

Both local anesthesia and general anesthesia were reliable for PKP. However, local anesthesia was more efficient and safer with less expense and more bearable pain when compared with general anesthesia.

## 1. Introduction

It is well known that vertebral compression fracture (VCF) can cause severe and long time pain [1], and it may lead to nerve injury, mental disease, and even disability without proper treatment [2]. The incidence of VCF has been increasing and the patients are becoming younger recently [3, 4]. VCF can also cause height loss and kyphosis, which always reduces the quality of life [5]. Long-term bedridden can even cause deadly hypostatic pneumonia and decubitus ulcer. Percutaneous kyphoplasty (PKP) is a good treatment for patients of VCF who cannot bear the pain or do not get well from conservative treatment [6]. Nowadays, both local anesthesia and general anesthesia are widely used in the PKP process [7–12]. However, the controversy about the best type of anesthesia for PKP has never been stopped. As far as we know, there were few studies focusing on this topic.

The purpose of this study is to find the best type of anesthesia for PKP.

## 2. Materials and Methods

The study was authorized by the Ethics Committee of the Second Hospital of Dalian Medical University (DMU).

### 2.1. Patient Population

PKP for all patients was performed at the First Operating Room of the Second Affiliated Hospital of DMU from Jan 2014 to Jan 2017. All data were retrospectively reviewed from the medical records and bills.

The inclusion criteria [13] were planned as follows:

1. The compression was over 15% of the height of the injured vertebra
2. Single-level VCF was diagnosed by doctors
3. The severe back pain had been treated by conservative treatments for 14 days before PKP, but not effective enough
4. The pain was over 5, measured by visual analogy score (VAS)
5. Percussion and tenderness on the posterior midline were detected
6. In magnetic resonance imaging (MRI), a hypointense signal on T1-weighted images was observed at the injured level
7. In MRI, a hyperintense signal on T2-weighted stir fat-suppressed images was observed at the injured level

The exclusion criteria [13] were planned as follows:

1. The fracture was caused by secondary osteoporosis
2. The patient got coagulopathy
3. The patient was in cachexia or ASA IV-V
4. There was no pain caused by VCF
5. The fracture was caused by metastatic cancer
6. There was a symptomatic neurologic injury

According to the inclusion and exclusion criteria, a total of 85 patients (45 patients who received local anesthesia were in group L and 40 patients with general anesthesia were in group G) were recruited in this study.

The demographic data of patients were collected one day before the operation from medical records at the ward. They included but are not limited to age, gender, body weight, height, body mass index (BMI), and smoking history. Injury mechanisms were divided into fall, traffic, sports, and others. Compensation was recorded according to the bills. The fracture level and operator were also collected.

### 2.2. Outcome Measures

The outcomes indicators were set in accordance with published research [13]. Clinical outcome was measured by operation time, severe complications, and VAS pain score of before, during, and after the operation. Operation time was obtained from anesthesia records. Severe complications consisted of myocardial ischemia, lung disease, and delirium.

Zero of VAS indicated no pain. Ten of VAS meant an ultimate pain. The VAS of patients was measured by a researcher who did not know this study. Anteroposterior and lateral radiographs were obtained before and after the operation. Vertebral height and kyphotic angle (KA) were calculated by measuring the radiographs as described in the published article [13]. Briefly, the posterior height (PH) of caudal vertebra under the injured level was set as 100%. Then, the anterior height (AH) and posterior height (PH) of the injured vertebra were calculated similarly and presented as percentage of PH. The KA was defined as an acute angle between the upper endplate of the head-end vertebra and the lower endplate of the tail-end vertebra.

### 2.3. Expenditures

Total expenditure and expenditures for anesthesia, device, drugs, and nursing were collected from medical bills of each patient. The medical expenditures outside of our institution were not involved. All participants declared that they had no extra medical expenditure outside of our institution from Jan 2014 to Jan 2017. Expenditures were collected 12 months after the operation. All expenditures were calculated as RMB.

### 2.4. Statistical Analysis

All data were analyzed by SPSS (Version 12, SPSS Cooperation, Chicago, IL). The classified variable was calculated by chi-square test and Fisher's exact test. They were shown as a figure with percentage. The continuous variable was calculated by Mann–Whitney test, paired or unpaired *t*-test with or without Welch's correction. Continuous variable was shown as mean ± standard deviation. All statistical results are presented as tables. *P* < 0.05 indicates the difference is statistically significant.

## 3. Results

### 3.1. Subject Characteristics

According to the inclusion and exclusion criteria, a total of 85 patients were included in this study (Table 1). 45 patients who received local anesthesia were recruited in group L and 40 patients undergoing general anesthesia were in group G. Table 1 presents the indicators of both groups at the baseline. The differences of indicators between the two groups were not significant (*P* > 0.05, all).

### 3.2. Clinical Results

The operation time and severe complications are shown in Table 1. The VAS of pain score before, during, and after PKP was also recorded (Table 2). The operation time in group L was significantly shorter than that in group G (*P* < 0.05). The incidence of severe complications in group L was significantly lower than that in group G (*P* < 0.05). Myocardial ischemia occurred in two patients with history of coronary heart disease during the operation in group G. One patient with history of asthma developed asthmatic attack just after intubation and recovered by spraying salbutamol aerosol and intravenous methylprednisolone in group G. Another patient of 80 years old developed delirium after the operation and got well 4 days later.

In both groups, the pain was significantly relieved after the operation when compared with that before the operation (*P* < 0.05). However, the degree of pain relief between the two groups had no significant difference (*P* > 0.05). There was no significant difference in VAS pain score before and after the operation between the two groups (*P* > 0.05). During the operation, the VAS pain score in group L was 2.939 ± 0.9934, while it could not be assessed in group G because of general anesthesia. However, after the operation, no patients said they feel pain in the period of the operation, so we still consider VAS pain score during the operation to be 0. Thus, the VAS pain score during the operation in group L was significantly higher than that in group G (*P* < 0.05).

### 3.3. Radiological Results

Radiological data were obtained as described above. AH and PH were analyzed (Table 3). AH KA in both groups was also compared (Table 4). All these radiological indicators showed there was no significant difference between the two groups at the same time point (*P* > 0.05, respectively). In the meantime, there was no significant difference in PH presented before and after the operation (*P* > 0.05). After the operation, AH in group L was significantly increased (94.10 ± 21.19) than that before the operation (80.92 ± 31.64) (*P* < 0.05), and AH in group G was significantly increased after the operation (93.17 ± 14.02) than that before the operation (80.10 ± 9.169) (*P* < 0.05). KA in group L was significantly decreased after the operation (6.344 ± 8.431) than that before the operation (12.04 ± 7.093) (*P* < 0.05), and KA in group G was significantly decreased after the operation (7.051 ± 4.711) than that before the operation (12.01 ± 3.183) (*P* < 0.05). The data shown above demonstrated that the PKP in both groups were effective on deformity correction.

### 3.4. Expenditures

**Figure 1:**
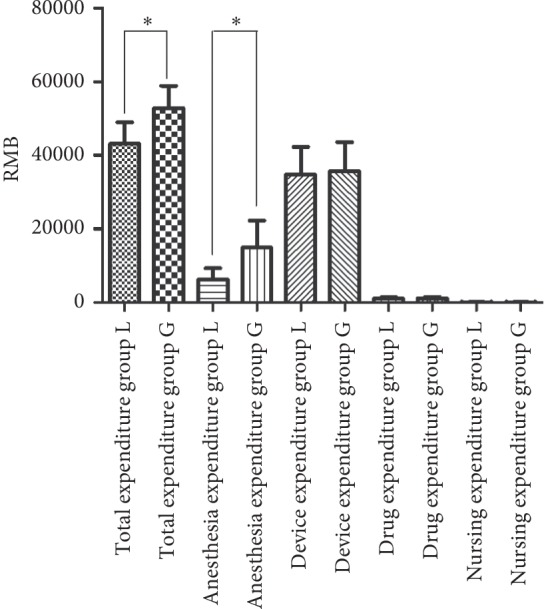
Comparison of expenditures between L group and G group. Error bars represent +1 SD from the mean. ^*∗*^Statistically significant (*P* < 0.05).

The expenditures of both groups are shown in Table 5 and Figure 1. In group L, total expenditure and anesthesia expenditure were significantly lower than those in group G (*P* < 0.05). There was no significant difference between the two groups when it came to device, drug, or nursing expenditures (*P* > 0.05).

## 4. Discussion

A desired method for the treatment of VCF should offer pain relief and a deformity correction fast and safely [14–16]. Percutaneous vertebroplasty (PVP) and PKP have been widely used recently, which can meet the needs of patients who want to relief the pain and correct the deformity [17, 18]. Some researches demonstrated that PVP and PKP had similar effects on pain killing and function improving [19–21]. However, recently published meta-analysis [22] showed that PKP has more merits compared with PVP. So, PKP should be recommended to people for the treatment of VCF.

However, there was still controversy about which kind of anesthesia was better for PKP as both local anesthesia and general anesthesia are used widely at present [11, 12]. In this study, patients with single-level VCF were included. Clinical outcomes, radiological outcomes, and expenditures were compared between local and general anesthesia.

Though the VAS pain score in group L during the operation is higher than that in group G, there are still several other reasons supporting the usage of local anesthesia for PKP. First, the pain of local anesthesia during the operation is relatively bearable (the VAS in L group is about 2.939, Table 2). Second, the sense of pain during the operation can be used as a protection for severe nerve injury because patients will give feedback to the operator when the nerve is going to be hurt. Third, the AH and KA between the two groups had no significant difference, which is in consistence with the previous published studies [23–26] and indicates that the type of anesthesia has no impact on the treatment effect of PKP. Therefore, local anesthesia, instead of general anesthesia, should be adopted for PKP for the treatment of VCF.

We also found more advantages in local anesthesia for PKP. The operation time in group L was shorter than that in group G. Group L needed less expenditure when compared with group G. More severe complications happened in group G such as myocardial ischemia and infection of the lung after the operation, while fewer happened in group L. This was also an important reason for high expenditure in group G too. According to the above data, local anesthesia showed its advantages, such as shorter operation time, lower incidence of severe complications, and less expenditure.

However, local anesthesia might not be good for all patients with VCF. In this study, we chose patients with single-level VCF which caused short operation time and less expenditure. While for multiple-level VCFs, general anesthesia may be a good choice because of the complicated operation and longer operation time and uncomfortable feeling of the prone position. So the anesthesia choice is relative and it should be planned by the patient's VCF condition and the patient's desire.

Expenditures can be divided into micro- and macrocosts [27, 28]. Macrocost focuses on the sum of the expenditures in a specific period. The merit of macrocost is that its data are easier to collect and calculate than that of microcost. But the details in the macrocost will be ignored, which is its internal drawback. In comparison, microcost lists all the items of the expenditures in a specific period, including the resources and the categories. So the expenditures of our study were collected and analyzed in the method of microcost.

According to the opinion of the published article [29], direct and indirect expenditures are supposed to be collected for cost analysis. However, the guidelines from the UK, Netherlands, and South Korea indicate that it is also acceptable to do the cost analysis with only direct expenditures [30, 31]. Therefore, we collected only direct expenditures in this study.

Referring to the published research [32], recommendations for medical procedures can be various from A to E. Grade A means the new procedure is cheaper and equally or more effective than the old one, which should be recommended strongly. Grade E means the new procedure is less or equally effective but more expensive, which should be rejected. The degrees of recommendations of grades B, C, and D are between A and E. The local anesthesia for PKP is supposed to be scored as grade A, which means the procedure should be strongly recommended.

There are still several points for consideration. Firstly, inherent limitations for retrospective study are not able to be avoided. Prospective studies should be better to verify the conclusions in the future. Secondly, the methods for appraising clinical outcomes such as cost-utility analysis were not applied in this study. Other researches focusing on this topic had better apply the cost-utility analysis to get a more affirmed conclusion [32]. Lastly, patients undergoing local anesthesia, who still felt pain and were nervous, should use conscious sedation, such as dexmedetomidine plus some opioids to make patients comfortable, which needs an anesthesiologist to keep patients safe and more expenditure.

## 5. Conclusions

PKP is an effective treatment for patients with VCFs. General anesthesia led to more serious complications, while local anesthesia was more effective, safer, and cost less. Therefore, although patients may endure tolerable pain, local anesthesia is more suitable for PKP for patients with single-level VCF when compared with general anesthesia.

## Figures and Tables

**Table 1:** Characteristics of the study population.

Characteristics	Group L (*n*=45)	Group G (*n*=40)	*P*	Statistical method
Age	73.43 ± 7.181	75.01 ± 9.653	0.4320	Mann–Whitney test
Male	25 (55.6)	19 (47.5)	0.5179	Fisher's exact test
Body mass index (kg/m^2^)	21.98 ± 2.511	22.99 ± 2.719	0.1109	Mann–Whitney test
Smoking	8 (17.8)	8 (20.0)	0.6123	Fisher's exact test
Injury mechanism			0.3596	Chi-square
Fall	29	21		
Traffic or sports injury	6	10		
Others	10	9		
Compensation	25 (55.6)	21 (0.525)	0.8295	Fisher's exact test
Fracture level			0.4778	Chi-square
T	15	10		
L	30	30		
Operator			0.4157	Chi-square
No. 1	11	10		
No. 2	16	18		
No. 3	9	6		
No. 4	7	2		
No. 5	2	4		
Operation time	40.89 ± 29.91	59.09 ± 21.11	0.0441	Mann–Whitney test
Severe complications	0	4	0.0451	Fisher's exact test

Data are presented as mean ± standard deviation and number (percentage values).

**Table 2:** Comparison of VAS pain scores before, during, and after the operation in group L and group G .

Group	VAS before the operation	VAS during the operation	VAS after the operation
Group L	7.332 ± 0.8761	2.939 ± 0.9934^a^	0.4472 ± 0.6121^b^
Group G	7.502 ± 0.9874	0^a^	0.4459 ± 0.7136^b^

Data are presented as mean ± standard deviation. VAS, Visual Analogue Scale. ^a^*P* < 0.05 when compared with preoperative VAS; ^b^*P* < 0.05 when compared with preoperative VAS.

**Table 3:** Comparison of anterior and posterior heights before and after the operation in L and G groups.

Group	AH before the operation	AH after the operation	PH before the operation	PH after the operation
Group L	80.92 ± 31.64	94.10 ± 21.19^a^	91.15 ± 16.99	93.17 ± 14.02
Group G	80.10 ± 9.169	90.98 ± 12.24^a^	89.91 ± 8.951	91.67 ± 9.714

Data are presented as mean ± standard deviation. AH, anterior height; PH, posterior height. ^a^*P* < 0.05 when compared with preoperative VAS.

**Table 4:** Comparison of kyphotic angles before and after operation in L and G groups.

Group	KA before the operation	KA after the operation	Change of KA
Group L	12.04 ± 7.093	6.344 ± 8.431^a^	3.504 ± 6.011
Group G	12.01 ± 3.183	7.051 ± 4.711^a^	3.772 ± 4.221

Data are presented as mean ± standard deviation. KA, kyphotic angle. ^a^*P* < 0.05 when compared with preoperative VAS.

**Table 5:** Comparison of expenditure in group L and group G .

Expenditure	Group L (*n*=45)	Group G (*n*=40)	*P*	Statistical method
Total expenditure (RMB)	43170 ± 5831	52920 ± 6012	**<0.0001**	Mann–Whitney test
Anesthesia expenditure (RMB)	6221 ± 3112	14989 ± 7231	**<0.0001**	Mann–Whitney test
Device expenditure (RMB)	34841 ± 7493	35773 ± 7804	0.1665	Mann–Whitney test
Drug expenditure (RMB)	1099 ± 399.5	1120 ± 509.5	0.3127	Mann–Whitney test
Nursing expenditure (RMB)	128.5 ± 39.55	132 ± 44.75	0.7091	Mann–Whitney test

Data are presented as mean ± standard deviation.

## Data Availability

The data used to support the findings of this study are included within the supplementary information files.

## Supplementary Materials

Supplementary Materials PKP for all patients was performed at the First Operating Room of the Second Affiliated Hospital of DMU from Jan 2014 to Jan 2017. All data were retrospectively reviewed from the medical records and bills. Research data include characteristics of the study population, comparison of VAS pain scores before, during, and after operation in L and G groups, comparison of anterior and posterior heights before and after the operation in L and G groups, comparison of kyphotic angles before and after the operation in L and G groups, comparison of expenditure in L and G groups. *Statistical Analysis.* All data were analyzed by SPSS (Version 12, SPSS cooperation, Chicago, IL). The classified variable was handled by chi-squared test and Fisher's exact test. They were shown as figure with percentage. The continuous variable was treated by Mann–Whitney test, paired or unpaired *t*-test with or without Welch's correction. The continuous variable was shown by mean ± standard deviation. All statistical approaches were presented by the tables of the article. *P* < 0.05 indicates the difference is statistically significant.
